# Dopamine- and Tyrosine Hydroxylase-Immunoreactive Neurons in the Brain of the American Cockroach, *Periplaneta americana*

**DOI:** 10.1371/journal.pone.0160531

**Published:** 2016-08-05

**Authors:** Yoshitaka Hamanaka, Run Minoura, Hiroshi Nishino, Toru Miura, Makoto Mizunami

**Affiliations:** 1 Faculty of Science, Hokkaido University, Sapporo, Hokkaido, Japan; 2 Laboratory of Ecological Genetics, Graduate School of Environmental Science, Hokkaido University, Sapporo, Hokkaido, Japan; 3 Research Institute for Electronic Science, Hokkaido University, Sapporo, Hokkaido, Japan; Tohoku University, JAPAN

## Abstract

The catecholamine dopamine plays several vital roles in the central nervous system of many species, but its neural mechanisms remain elusive. Detailed neuroanatomical characterization of dopamine neurons is a prerequisite for elucidating dopamine’s actions in the brain. In the present study, we investigated the distribution of dopaminergic neurons in the brain of the American cockroach, *Periplaneta americana*, using two antisera: 1) an antiserum against dopamine, and 2) an antiserum against tyrosine hydroxylase (TH, an enzyme required for dopamine synthesis), and identified about 250 putatively dopaminergic neurons. The patterns of dopamine- and TH-immunoreactive neurons were strikingly similar, suggesting that both antisera recognize the same sets of “dopaminergic” neurons. The dopamine and TH antibodies intensively or moderately immunolabeled prominent brain neuropils, e.g. the mushroom body (memory center), antennal lobe (first-order olfactory center) and central complex (motor coordination center). All subdivisions of the mushroom body exhibit both dopamine and TH immunoreactivity. Comparison of immunolabeled neurons with those filled by dye injection revealed that a group of immunolabeled neurons with cell bodies near the calyx projects into a distal region of the vertical lobe, which is a plausible site for olfactory memory formation in insects. In the antennal lobe, ordinary glomeruli as well as macroglomeruli exhibit both dopamine and TH immunoreactivity. It is noteworthy that the dopamine antiserum labeled tiny granular structures inside the glomeruli whereas the TH antiserum labeled processes in the marginal regions of the glomeruli, suggesting a different origin. In the central complex, all subdivisions excluding part of the noduli and protocerebral bridge exhibit both dopamine and TH immunoreactivity. These anatomical findings will accelerate our understanding of dopaminergic systems, specifically in neural circuits underlying aversive memory formation and arousal, in insects.

## Introduction

Biogenic amines have been anatomically and biochemically identified in the central nervous system of vertebrates as well as invertebrates. In insects, these molecules regulate various physiological phenomena as neurotransmitters, neuromodulators, or neurohormones [1,2]. Among these, the catecholamine dopamine has been implicated in important roles in regulating motor behavior [1], caffeine-induced arousal [3], circadian entrainment [4], and learning and memory [5–7]. Besides these, dopamine may be involved in additional functions, considering its widespread distribution in central nervous systems [8–12]. However, the cellular and molecular basis of dopamine’s actions have not been fully elucidated in any insect species. One system, in which the subcellular effects of dopamine have been most intensively studied, is the cockroach salivary gland [13–17].

Dopamine is synthesized from the amino acid tyrosine, the latter being converted into DOPA by tyrosine hydroxylase (TH), a rate-limiting enzyme in dopamine biosynthesis. DOPA is further converted into dopamine by DOPA decarboxylase (DDC) [1,18]. Recent advances in molecular biology have allowed the characterization of the structure and physiological roles of insect dopamine receptors, especially in the fruit fly, *Drosophila melanogaster*, honey bee, *Apis mellifera*, and cricket, *Gryllus bimaculatus* [19–21]. All dopamine receptors in insects belong to the G-protein-coupled receptor (GPCR) superfamily. So far, four different types of dopamine receptors have been cloned: 1) D1-like dopamine receptors (Dop1); 2) Invertebrate dopamine receptors (INDR; 3) D2-like dopamine receptors (Dop3); and 4) dopamine/ecdysteroid receptors (DopEcR) [19]. The D1-like receptors are coupled to G_αs_ proteins, which in turn activate adenylyl cyclase to raise intracellular cAMP concentrations. In contrast, the D2-like receptors are coupled to G_αi_ proteins and inhibit adenylyl cyclase, or coupled to different intracellular second-messenger systems [22]. The INDRs activate adenylyl cyclase, and they are also coupled to Ca^2+^ signaling pathways [21]. The DopEcRs activated by dopamine increase cAMP concentrations and also activate the phosphoinositide 3-kinase pathway [23]. Despite this rich knowledge of dopamine receptors, we still have little information about the functional roles of dopamine itself. To address the neural basis of dopamine actions in insect brains, a detailed neuroanatomical mapping of putatively dopaminergic neurons, those thought to contain dopamine and therefore to release it physiologically, is indispensable.

Comprehensive neuroanatomical studies of putatively dopaminergic neurons exist in flies [10,12], the honeybee [9], and the desert locust [11]. In contrast, only some sets of neurons have been reported in the cockroach [24–26]. The cockroach has been used as an experimental object to study various brain functions, including the regulation of circadian rhythms by clock neurons [27–30], the processing of olfactory information by the antennal lobe [31–33], learning and memory tasks by the mushroom body [34–36], and locomotor control by the central complex [37–39]. In order to extend these studies further, we need a detailed anatomical description of putatively dopaminergic neurons in the cockroach. For this purpose, we used two antisera: 1) an antiserum raised against dopamine itself, and 2) an antiserum against its synthetic enzyme, TH. In the present account, we tentatively refer to neurons which are immunolabeled by the anti-dopamine and/or the anti-TH antiserum as “dopaminergic”.

The cockroach, *Periplaneta americana* is amenable to behavioral experiments including various forms of learning paradigm [35,36,40–46], and also because of its large brain size and ready accessibility to neurons to physiological studies such as microsurgery and electrophysiology [32–34,47]. Immunocytochemical analysis with antisera against dopamine and one of its synthesizing enzyme is a prerequisite for future neurophysiological studies of dopaminergic neurons. Here, we present the first comprehensive map of putatively dopaminergic neurons in the cockroach brain.

## Materials and Methods

### Animals

**Fig 1:**
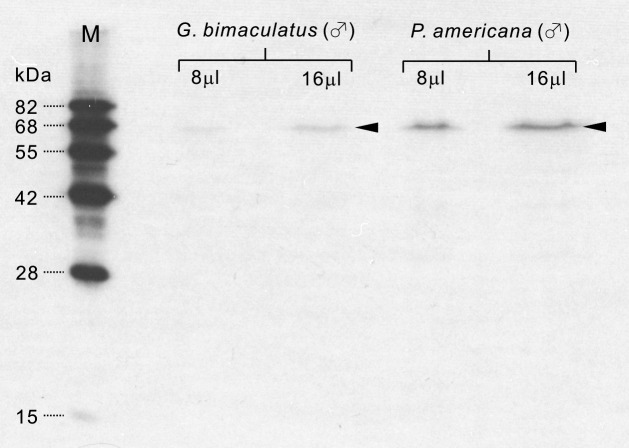
Immunoblots of tyrosine hydroxylase in the cricket and cockroach brain. Protein extracts (8 μl and 16 μl) from the male cricket, *Gryllus bimaculatus* and male cockroach, *Periplaneta americana* were electrophoresed, transferred to a membrane and reacted with anti-tyrosine hydroxylase (TH). The anti-TH recognized a single band of protein of ~ 66 kDa (arrow heads) in both the cricket (left two lanes) and cockroach (right two lanes). M, molecular marker.

American cockroaches (*Periplaneta americana*) were raised in crowded colonies at Hokkaido University (Sapporo, Hokkaido, Japan) under a LD 12:12 h photoperiod at 27°C. Adult cockroaches of either sex were used for reduced silver impregnation while only adult males were used for immunocytochemistry. Male field crickets, *Gryllus bimaculatus* maintained under the same photoperiodic and temperature conditions at Hokkaido University were also used to test the specificity of an antiserum against tyrosine hydroxylase, as in Fig 1.

### Reduced silver impregnation

The head capsules were partially opened and immersed in cockroach saline containing 3% paraformaldehyde (PFA) for 1 hour, and then brains were carefully dissected out. The dissected brains were fixed in a solution containing 4% PFA, 5% glacial acetic acid and 85% ethanol for 2 days, dehydrated, and then embedded in paraffin. Reduced silver staining was performed on 12-μm sections, as described elsewhere [48].

### Mass injection of Neurobiotin

Cockroaches with their wings removed were mounted on a plastic dish. A large window was made in the cuticle of the anterior side of the head, and the tracheae and fat bodies removed to expose the vertical lobes of the mushroom bodies. After removing hemolymph with a piece of Kimwipe, a glass microelectrode with crystals of Neurobiotin at the tip was stabbed into the vertical lobe under a dissecting microscope. The exposed brain region was then briefly washed with saline. The window was resealed with the excised cuticle and covered with a piece of Kimwipe soaked in saline, and then the cockroach left overnight at 4°C to allow the injected Neurobiotin to be taken up by neurites damaged by the microelectrode.

### Visualization of Neurobiotin-filled neurons

The Neurobiotin-injected tissues were fixed overnight at 4°C with 4% PFA and 1% glutaraldehyde (GA) in 0.067 M phosphate buffer (PB, pH 7.4) containing 1% saturated picric acid. After fixation, the brain was dissected and embedded in a gelatin/albumin mixture (4.8% gelatin and 28% albumin in distilled water) and then post-fixed overnight at 4°C in 7–8% PFA in PB. The post-fixed tissues were sectioned in a frontal plane at a thickness of 40 μm with a vibrating blade microtome, then washed in PB, and incubated overnight at 4°C with streptavidin-horseradish peroxidase (HRP, 1:200; RPN1231, Amersham Biosciences, Bucks, UK) in 0.01 M phosphate-buffered saline (PBS, pH 7.4) containing 0.5% Triton X (PBST). The sections were developed with a solution of 0.032% 3,3’-diaminobenzidine tetrahydrochloride (DAB) in 0.1M Tris-HCl (pH 7.4) containing 0.0145% H_2_O_2_ and 0.3% nickel ammonium sulfate. After washing in PB, the sections were mounted on a gelatin-coated glass slide, dehydrated in a graded ethanol series, cleared in xylene, and mounted in Mount-Quick (Daido Sangyo, Tokyo, Japan) beneath a cover slip.

### Antibody characterization

For anti-dopamine immunolabeling, a rabbit polyclonal antiserum against dopamine-bovine serum albumin (BSA)-GA conjugate (cat. No. AB122S) was purchased from Millipore (Temecula, CA, USA; Table 1). According to the manufacturer’s data sheet, the antibody specifically labels dopaminergic neurons of substantia nigra and of the A10 region as well as dopaminergic nerve terminals of the mammalian locus coeruleus. Immunolabeling in these areas is abolished by pre-incubation with 10–100 μg dopamine-BSA-GA conjugate per ml of diluted antibody. By contrast, repeated absorptions of the serum with noradrenaline coupled to BSA does not alter the immunocytochemical labeling pattern. We confirmed the specificity of the anti-dopamine antiserum in the cockroach by liquid-phase preadsorption. Working dilutions of the antiserum, which were preadsorbed with conjugates of dopamine coupled with GA to BSA, were applied to sections of *P*. *americana* brains. Preadsorption with 0.4 μM or more of the BSA-dopamine conjugate abolished immunoreactive signals.

**Table 1:** Antibodies used for immunocytochemistry.

Antibody	Immunogen	Source	Working dilution
Anti-dopamine	Dopamine-glutaraldehyde-bovine serum albumin conjugate	Rabbit polyclonal, Cat. No. AB122S, Millipore	1:2000
Anti-synapsin	synapsin protein coupled to glutathione-S-transferase	Mouse monoclonal, 3C11, Developmental Studies Hybridoma Bank	1:50
Anti-tyrosine hydroxylase	SDS-denatured rat tyrosine hydroxylase	Rabbit polyclonal, Cat. No. NB300-109, Novus biologicals	1:2000–1:4000 (DAB) 1:2000 (fluorescence)

For anti-tyrosine hydroxylase (TH) immunolabeling, a rabbit polyclonal antiserum raised against SDS-denatured rat TH (Cat. No. NB300-109) was purchased from Novus biologicals (Littleton, CO, USA; Table 1). According to the data sheet supplied by the manufacturer, the TH antibody immunolabels dopamine neurons in the mouse substantia nigra, and also specifically detects a 60 kDa TH protein in rat caudate lysate. We demonstrated specificity of the anti-TH in *P*. *americana* brains and also in the brains of the male cricket *G*. *bimaculatus* by standard procedures for Western blots as described below. The anti-TH detected proteins of ~ 66 kDa in brain homogenates of both *P*. *americana* and *G*. *bimaculatus* (Fig 1), which are slightly larger than the molecular size for *Drosophila melanogaster* TH (58 kDa) [49].

For immunolabeling neuropil structures, a mouse monoclonal antibody against *D*. *melanogaster* synapsin (SYNORF1 or antibody 3C11, Developmental Studies Hybridoma Bank, Iowa City, IA) was used (Table 1). Synapsins belong to a small family of synaptic vesicle-associated phosphoproteins that participate in regulating transmitter release [50]. Anti-synapsin detects a broad band of proteins of ≈ 80 kDa in cockroach brain homogenates [51], which corresponds to a triplet of *D*. *melanogaster* synapsin isoforms (of 70, 74, and 80 kDa, respectively) [52].

### Immunoblotting

Sodium dodecylsulfate polyacrylamide gel electrophoresis (SDS-PAGE) was performed in 4.5% stacking and 12% running gel. Cockroach or cricket brains were dissected out in PBS. After dissection, two brains were transferred into 50 μl of lysis buffer (57.7 mM Tris-base, 10% glycerine, and 2% SDS) containing 1.5 μl ß-mercaptoethanol (Z523A, Promega, WI, USA), and immediately homogenized. The homogenate (containing two brains) was heated for 3 min at 100°C, transferred on ice and then centrifuged. The supernatant was subjected to SDS-PAGE and blotted on a Hybond-P PVDF membrane (RPN303F, GE Healthcare UK Ltd, Little Chalfont, Bucks, UK). As a molecular weight marker, Dr. Western (Oriental Yeast Co., Ltd, Tokyo, Japan) was used. After blocking the membrane with 5% skim milk in Tris-buffered saline containing 0.1% Tween 20 (TBST, pH 7.6) for 30 min, it was incubated with the rabbit anti-tyrosine hydroxylase (1: 5,000) in TBST for 2 h at 25°C. The membrane was rinsed in four changes of TBST, and incubated in donkey anti-rabbit IgG conjugated to HRP (1:2,000; NA934V, GE Healthcare UK Ltd) in TBST for 90 min at 25°C. After being rinsed in four changes of TBST, the ECL Western Blotting Starter Kit (RPN2108, GE Healthcare UK Ltd) was used to visualize immunoreactivity.

### Immunofluorescent labeling

For dopamine immuno-labeling, brains were fixed with 2.5% GA and 1% sodium metabisulfite (SMB) in PB. For tyrosine hydroxylase immunolabeling, brains were fixed with 4% PFA in PB overnight at 4°C.

The fixed tissues were embedded in a gelatin/albumin mixture, post-fixed, and sectioned as described above (0.5% SMB was added to the fixative for dopamine labeling). Non-specific binding sites were blocked with 5% normal goat serum (NGS) in 0.01 M PBST for 1 hour, and then incubated with respective primary antibodies at working dilutions in PBST containing 5% NGS listed in Table 1 for 2 days at 4°C. For dopamine labeling, sections were incubated with 0.25% sodium borohydride in PB, before blocking process. To visualize neuropil structures, sections were simultaneously incubated with the mouse anti-synapsin antibody. After washing in PBST, the sections were incubated in secondary antibodies (anti-rabbit IgG conjugated to Cy3 (1:200; Jackson ImmunoResearch, West Grove, PA, USA) and goat anti-mouse IgG conjugated to Alexa Fluor 488 (1:200; Molecular Probes, Eugene, OR, USA)) in PBST containing 5% NGS overnight at 4°C, followed by washes in PBST and PBS. The sections were mounted beneath cover slips in Vectashield (H-1000; Vector, Burlingame, CA, USA).

### Immunoperoxidase labeling

For dopamine and TH immunolabeling, we also applied an immunoperoxidase labeling method. Procedures prior to the secondary antibody incubation were as described above. After primary antibody incubation, the sections were incubated in goat anti-rabbit IgG conjugated to HRP (1:200, Jackson ImmunoResearch) in PBST containing 5% NGS. After washing in PBST, sections were developed, mounted on glass slide, dehydrated, cleared and then mounted in Mount-Quick beneath a cover slip as described above.

### Photography and tracing

DAB-labeled and reduced silver stained preparations were imaged with a digital camera (DMC-G5, Panasonic, Osaka, Japan) mounted on a compound microscope (BX60, Olympus, Tokyo, Japan). Several images at different focal planes were manually captured from single sections at intervals of 3–5 μm, and then superimposed into a single image with Zerene Stacker software (Zerene Systems LLC, WA, USA).

Sections labeled by immunofluorescence were imaged using a Zeiss LSM510 confocal microscope (Carl Zeiss, Jena, Germany) equipped with Plan-Apochromat 20X/0.75 (Carl Zeiss). Alexa 488 was excited with an Ar laser at 488 nm and viewed through a 475-525-nm band-pass filter, and Cy3 was excited with a HeNe green laser at 543 nm through a 560-nm long pass filter. Confocal images were acquired at z-axis intervals of 1.5 to 2 μm and a resolution of 1,024 x 1,024 pixels. All confocal images are compressed from stacks of 10 to 15 optical sections. The size, contrast, and brightness of the images were adjusted using Photoshop CS4 (Adobe Systems, Tokyo, Japan) and Corel Draw X4 (Corel, Ottawa, ON, Canada).

## Results

**Fig 2:**
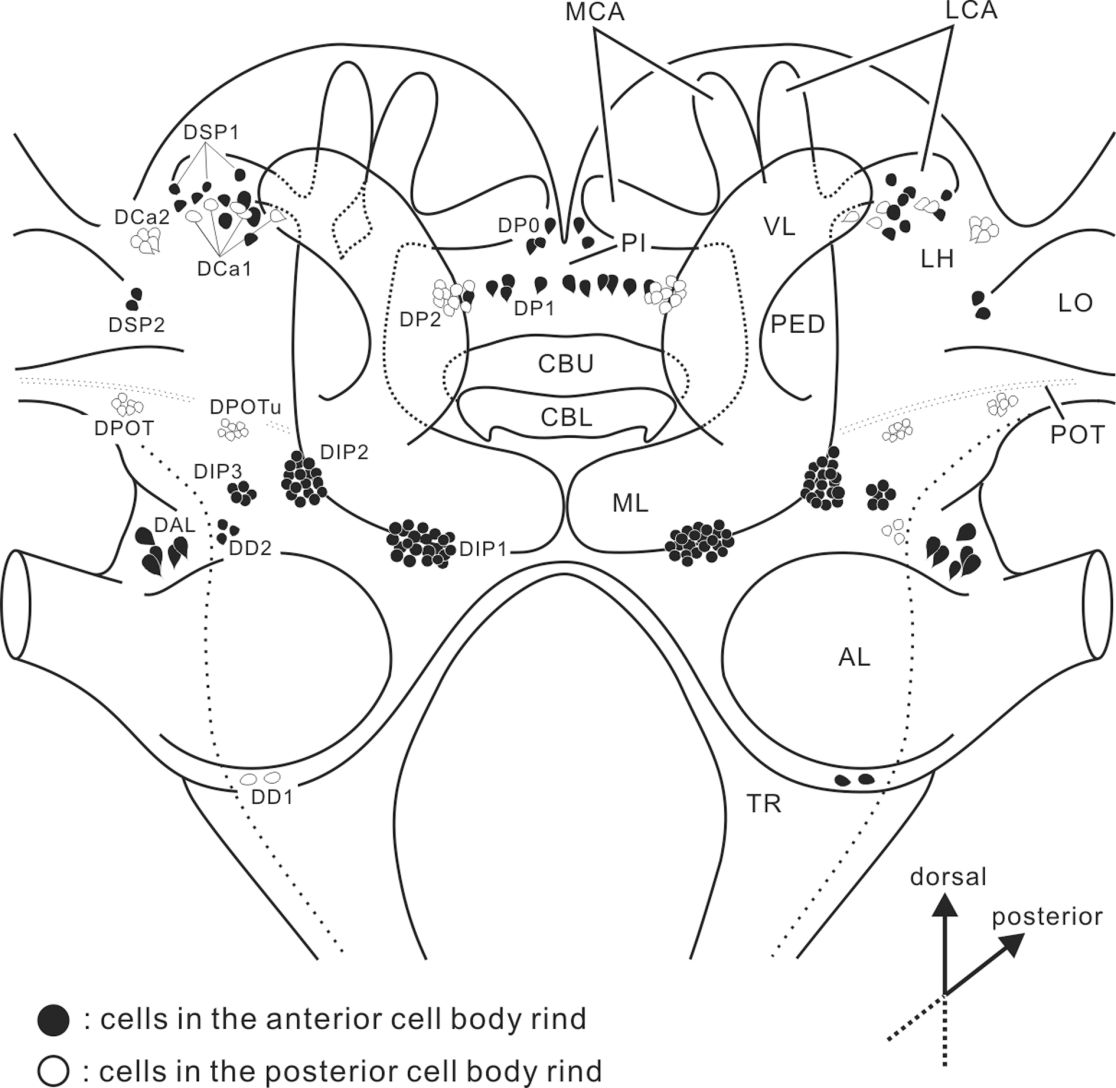
Diagram in a frontal plane showing the distribution of dopamine-immunoreactive cell bodies in the brain of the cockroach, *Periplaneta americana*. Cell bodies in the anterior cell body rind in the brain are shown in black while those in the posterior are in outline. Most are clustered: DP0-2, three groups in the pars intercerebralis (PI); DCa1-2; two groups lateral to the calyx; DSP1-2, two groups in the superior lateral protocerebrum; DIP1-3, three groups in the inferior protocerebrum; DAL, a group near the antennal lobe (AL); DD1-2, two groups in the deutocerebrum; DPOT, a group near the posterior optic tract (POT); DPOTu, a group near the posterior optic tubercle. CBL, central body lower division; CBU, central body upper division; LCA, lateral calyx; MCA, medial calyx; ML, medial lobe; PED, pedunculus; TR, tritocerebrum; VL, vertical lobe.

Immunoreactivity to an antiserum against dopamine was detected in all major neuropils of the cockroach brain except part of the central complex. In the brain, 15 different groups of cell clusters exhibit moderate to strong immunoreactivity (Fig 2 and Table 2). The number of cell bodies for each group is shown in Table 2. In the protocerebrum, three cell groups are located in the pars intercerebralis (DP0-2), two groups lateral to the calyx of the mushroom body (DCa1, DCa2), two cell groups in the superior lateral protocerebrum (DSP1, DSP2), three cell groups in the inferior protocerebrum (DIP1-3), a cluster ventral to the posterior optic tract (DPOT), and another cluster near the posterior optic tubercle (DPOTu). In the deutocerebrum, three cell groups exhibit dopamine immunoreactivity (DAL, DD1, DD2). In addition to anti-dopamine, we also employed an antiserum against tyrosine hydroxylase (TH), which is expressed exclusively in dopaminergic neurons in the *D*. *melanogaster* brains [12]. Both antisera immunolabeled almost the same populations of neurons in the brain, supporting the fact that the immunoreactive neurons synthesize dopamine and the specificity of both antibodies. Not only were both antisera raised in rabbits but each antiserum also requires a different fixative, so that double labeling experiments would have been difficult to achieve. We thus independently immunolabeled specimens with each antiserum to compare the both labeling patterns for details between specimens. In the present study, we analyzed 9 brains immunolabeled with the anti-dopamine and 24 brains immunolabeled with the anti-TH.

**Table 2:** Location of dopaminergic cell groups in the brain of *Periplaneta Americana*.

Cell group	*Number of cell bodies per hemisphere	Location of cell	Projections
DP0	4 (3)	anterior pars intercerebralis	ventrolateral protocerebrum, deutocerebrum and tritocerebrum
DP1	9 (22)	intermediate pars intercerebralis	?
DP2	46 (59)	posterior to the protocerebral bridge	central body and lateral accessory lobe
DCa1	21 (25)	ventro-lateral to the calyx	vertical lobe and superior lateral protocerebrum (?)
DCa2	10 (10)	lateral to the calyx and dorsal to the lobula	?
DSP1	24 (20)	superior lateral protocerebrum, lateral to the vertical lobe	?
DSP2	2 (2)	superior lateral protocerebrum	?
DIP1	40 (69)	inferior medial protocerebrum	superior medial protocerebral neuropil and medial lobe (?)
DIP2	42 (38)	inferior protocerebrum	central body and junction between pedunculus and lobes (?)
DIP3	9 (10)	inferior lateral protocerebrum	?
DPOT	7 (6)	ventral to the posterior optic tract	?
DPOTu	14 (20)	lateral to the posterior optic tubercle	posterior optic tubercle (?)
^†^DAL	• 13 (0) • 0 (1)	• dorsal to the antennal lobe • dorsal to the antennal lobe	• Local interneuron: antennal lobe • Projection neuron: antennal lobe, calyx and lateral horn (?)
DD1	3 (4)	deutocerebrum, ventral to the antennal lobe	?
DD2	4 (12)	posterior deutocerebrum	?

*Maximum number of dopamine-positive cells is shown, and that of tyrosine hydroxylase-positive cells is in parenthesis.

^†^DAL includes a single projection neuron and ~13 antennal lobe local interneurons.

### Dopamine immunoreactivity in the mushroom body

**Fig 3:**
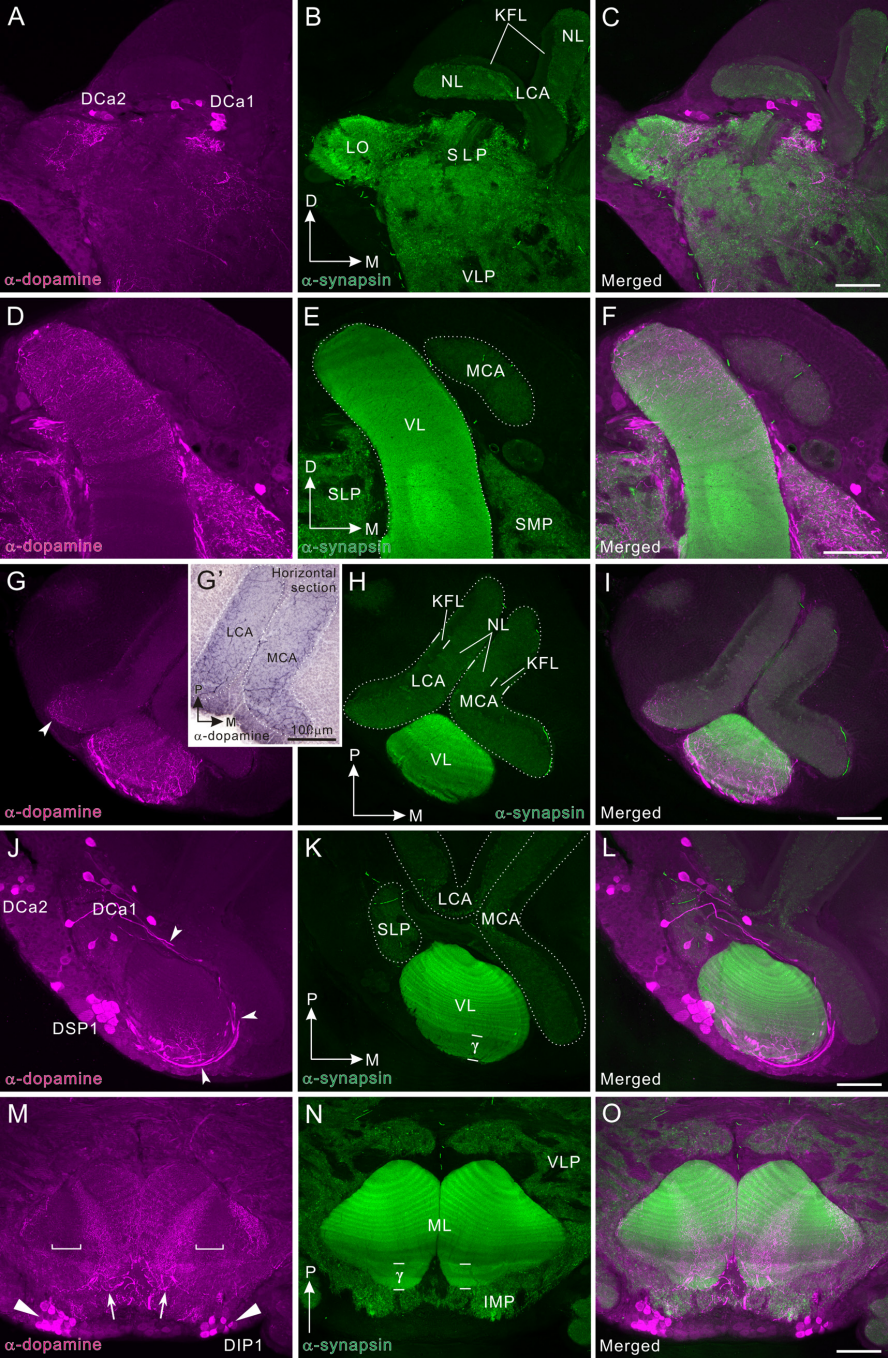
Dopamine-immunoreactivity in the mushroom body. All panels show a right brain hemisphere (dorsal to the top, medial to the right), except M-O (posterior to the top). Dopamine immunoreactivity is shown in magenta (A, D, G, J, M) and synapsin immunoreactivity in green (B, E, H, K, N). Corresponding merged images in C, F, I, L, O. **A-C**: Frontal sections through the lateral protocerebrum. DCa1 and 2 clusters are visible. **D-F**: Frontal sections through the vertical lobe. The distal part of the vertical lobe (VL) as well as the superior protocerebral neuropil are innervated by numerous dopamine-immunoreactive fibers. **G-I**: Horizontal sections through the tip of the vertical lobe, where dopamine-positive fibers are widely distributed. Dopamine-positive fibers in the calyx were faintly labeled by fluorescent immunolabeling technique (arrowheads in G) while those fibers were visualized by the HRP-mediated technique (G’), possibly because of the low content of dopamine. **J-L**: Horizontal sections through the calyx, ventral to panels G-I. DCa1 cell bodies project axons toward the anterior face of the vertical lobe bypassing the medial margin (arrowheads). These axons bear small fibers that invade layers of the vertical lobes that are immunolabeled by a synapsin antibody. DSP1 cell bodies are clustered in a region lateral to the vertical lobe. The anteriormost layer (γ layer) is predominantely innervated by immunoreactive fibers. **M-O**: Horizontal sections through the medial lobes (MLs). A band of dopamine-immunoreactive fibers invades the tip of the medial lobe parpendicularly to synapsin-positive layers (arrows). In a very marginal region around the midline, dopamine-positive fibers follow synapsin-positive layers. The DIP1 cell clusters are located in the inferior medial protocerebrum (IMP) anterior to the medial lobe (triangles). D, dorsal; KFL, Kenyon fiber layer; LCA, lateral calyx; LO, lobula; M, medial; MCA, medial calyx; NL, neuropil layer; P, posterior; SMP, superior medial protocerebrum; γ, γ layer; VLP, ventro-lateral protocerebrum. Scale bars = 100 μm.

**Fig 4:**
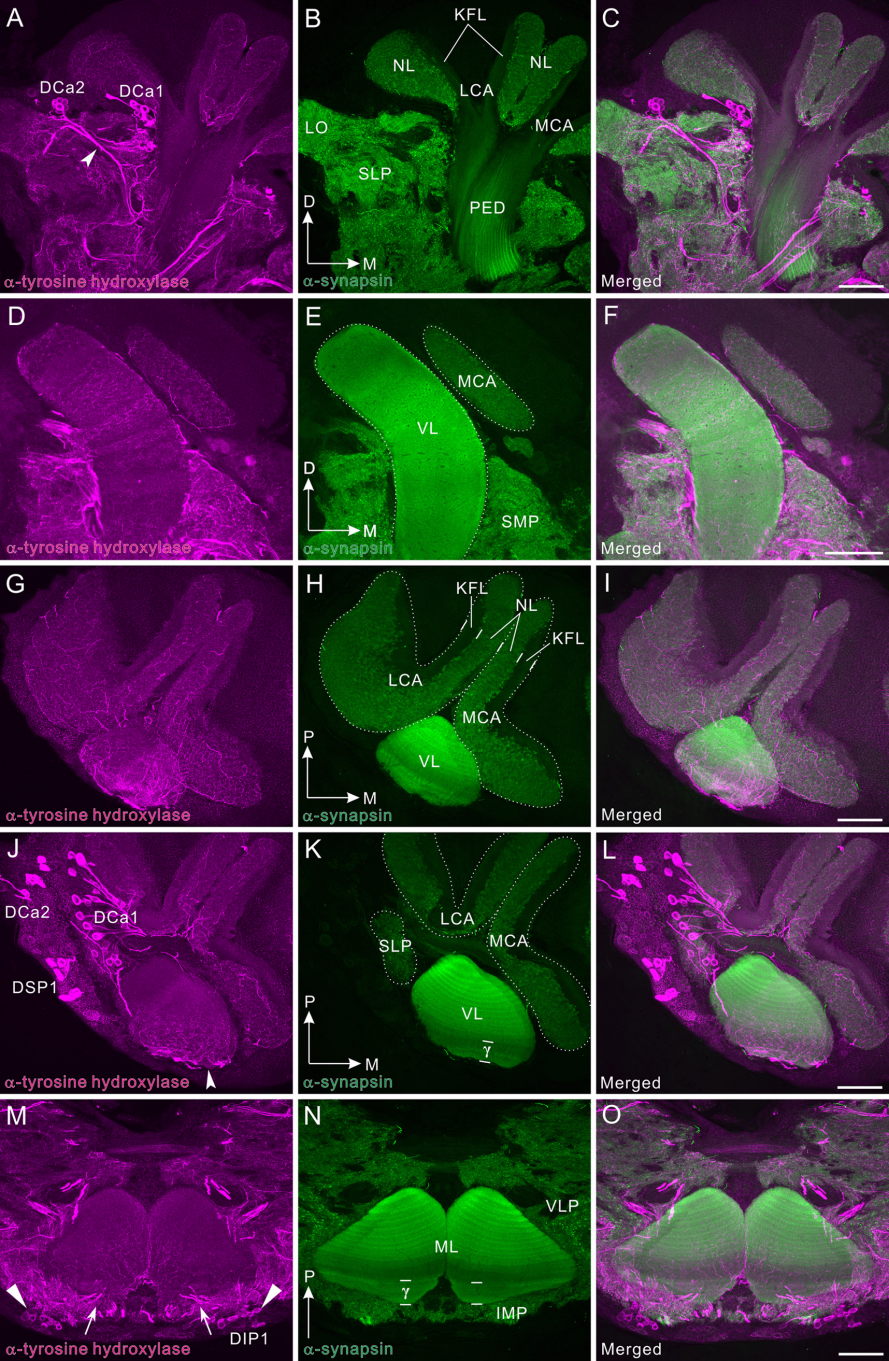
Tyrosine hydroxylase immunoreactivity in the mushroom body. All panels show a right brain hemisphere (dorsal to the top, medial to the right), except panels M-O (posterior to the top). Tyrosine hydroxylase (TH) immunoreactivity is shown in magenta (A, D, G, J, M) and synapsin immunoreactivity in green (B, E, H, K, N). Corresponding merged images in C, F, I, L, O. **A-C**: Frontal sections through the lateral protocerebrum. Two groups of TH-positive cell bodies (DCa1 and 2) were labeled. DCa2 appears to send the axons ventrally (arrowhead). The superior lateral protocerebrum (SLP) as well as the medial and lateral calyces (MCA and LCA, respectively) are innervated by TH-positive fibers. **D-F**: Frontal sections through the vertical lobe (VL) and parts of the medial calyx (MCA), which receive many fine TH-positive fibers. **G-I**: Horizontal sections through a tip of the vertical lobe and dorsal parts of the calyces. Both calyces and the vertical lobe are innervated by fibrous TH-positive fibers. Note that TH-immunoreactive fibers exclusively innervate synapsin-positive neuropil layers (NLs) of the calyx where the dendrites of Kenyon cells receive synaptic inputs from antennal-lobe projection neurons. **J-L**: Horizontal sections through the calyces, ventral to panels G-I. Here, three clusters of TH-positive cell bodies (DCa1, DCa2, and DSP1) are visible. Immunoreactive fibers mainly invade anterior layers including the γ layer (γ) in the vertical lobe. **M-O**: Horizontal sections through the distal portions of the medial lobes (MLs), invaded by fine TH-positive fibers exhibiting moderate immunolabeling (arrows). In the inferior medial protocerebrum (IMP) anterior to the medial lobe, a pair of DIP1 clusters (triangles) is situated. D, dorsal; KFL, Kenyon fiber layer; LCA, lateral calyx; LO, lobula; M, medial; P, posterior; PED, pedunculus; SMP, superior medial protocerebrum. Scale bars = 100 μm.

**Fig 5:**
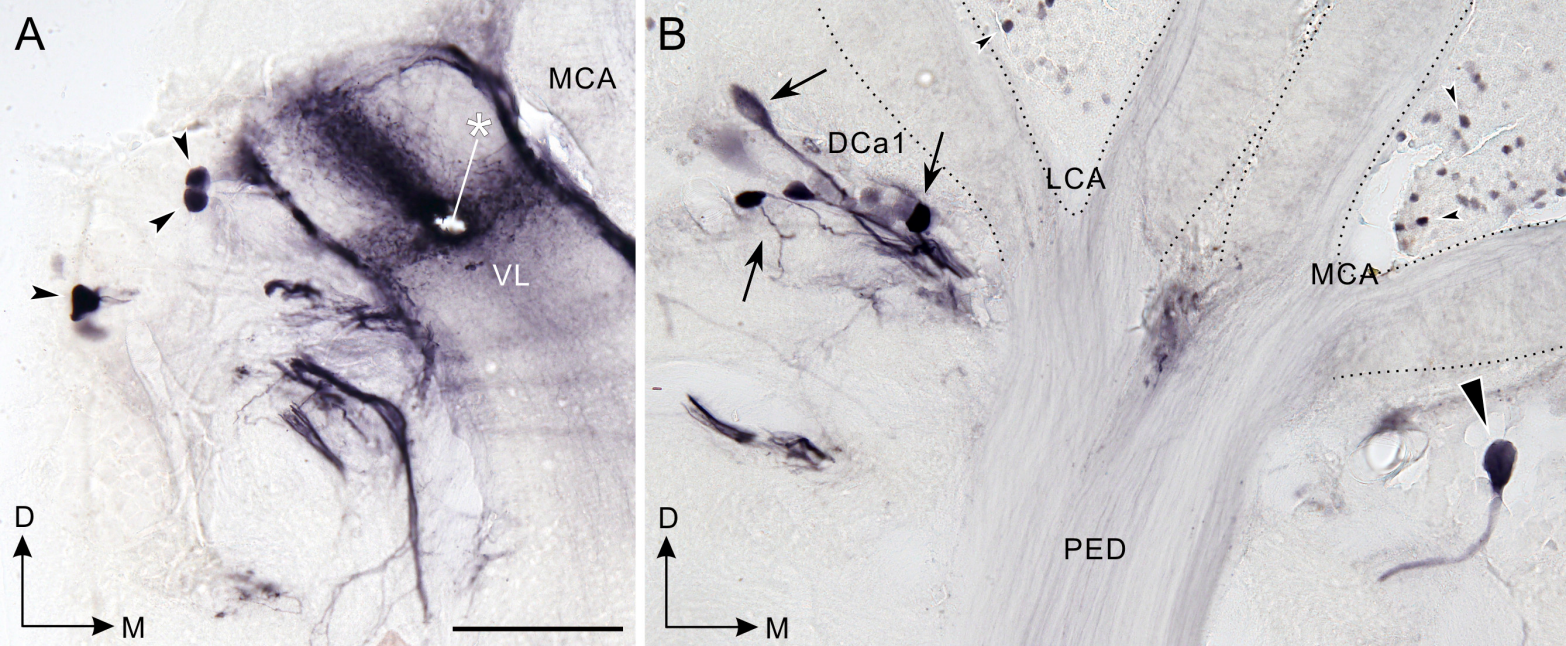
Neurons filled by injecting Neurobiotin into a distal area of the vertical lobe. Four different groups of cell bodies were successfully filled with the dye. **A, B:** Frontal sections through the vertical lobe (VL, A) and the calyx (B) (dorsal to the top, medial to the right). An asterisk indicates the site of injection. The first group of cell bodies is located in a region lateral to the vertical lobe (arrowheads), and the second group of cell bodies is near the lateral calyx (LCA, arrows). A single neuron is situated in a region ventral to the medial calyx (MCA, triangle). The fourth group are cell bodies of Kenyon cells (small arrows in B). D, dorsal; M, medial; PED, pedunculus. Scale bar = 100 μm (also applies to B).

The mushroom bodies of insects are paired neuropil structures, located in the central brain and comprising intrinsic neurons, termed Kenyon cells. The mushroom body of *P*. *americana* consists of several divisions as in other insects, i.e. paired lobes (vertical lobe and medial lobe), paired calyces (medial calyx and lateral calyx), and a pedunculus, (Fig 2). All of these neuropils are innervated by meshworks of dopamine-immunoreactive fibers (Fig 3). In the vertical and medial lobes, dopamine-immunoreactive fibers form several bands along the proximo-distal regions, and each band can be contributed by a distinct class of neurons (Fig 3D, 3G and 3M). It was noteworthy that in the medial lobe a part of the distal region is free of immunoreactivity (bracket in Fig 3M). This is also obvious in frontal sections. The distal medial lobe seems to be innervated in two different fashions. In a region medial to an immunonegaitve area (bracket in Fig 3M), a narrow band of fine arborizations (arrows in Fig 3M) invades the medial lobe perpendicularly to the layers called slabs or laminae [48,53], which are visualized by anti-synapsin. On the other hand, in the very marginal area around the midline, the dopamine-positive fibers appear to follow the synapsin-labeled layers (Fig 3M). Each slab or lamina representing axons of a different set of Kenyon cell types [54] is either intensely or weakly synapsin-immunolabeled. The anteriormost slab or lamina (the γ layer) corresponding to the γ lobe in the fruit fly [55], which consists of the axons of class II or K4 Kenyon cells [53,54], is visible (γ in Fig 3K and 3N), and innervated by dopamine-positive fibers. A similar pattern of synapsin labeling is also seen in Fig 4K and 4N. Dopamine-immunoreactive fibers in the vertical lobe, originate at least in part in DCa1 neurons having cell bodies beneath the lateral calyx (Fig 3A). The axons of DCa1 neurons loop medially along the surface of the vertical lobe and enter the lobe from the anterior surface to give rise to fine arborizations predominantly in distal areas (arrowheads in Fig 3J). This pattern of innervation was confirmed by dye injection experiments into the vertical lobe (Fig 5). In contrast to the lobes, the dopamine contents in the calyces appear to be low (Fig 3G and 3J). The more sensitive technique mediated by HRP did however label fibrous dopamine-immunoreactive processes in the medial and lateral calyces (Fig 3G’). More dopamine-positive fibers are visible in Kenyon fiber layer than in neuropil layer. Part of the pedunculus also receives dopamine-positive fibers (see below).

### Tyrosine hydroxylase immunoreactivity in the mushroom body

Tyrosine hydroxylase (TH) immunoreactivity presented a fibrous appearance in the mushroom body. TH immunolabeling also provided moderate immunoreactive signals as in the dopamine immunolabeling. The locations of TH-positive cell clusters (Fig 4) are strikingly similar to those of dopamine-positive cell clusters (Fig 3), confirming that both antisera recognize the same sets of neurons (e.g. DCa1/2 in Fig 4A vs DCa1/2 in Fig 3A; DSP1 in Fig 4J vs DSP1 in Fig 3J). A dense meshwork of TH-immunoreactive processes innervates the lateral and medial calyces (Fig 4A and 4G). Each calyx of the cockroach is divided into the neuropil layer (NL) and the Kenyon fiber layer (KFL, Figs 3B, 4B and 4H) [54,56]. TH-immunoreactive fibers predominantly distribute in the neuropil layer, which is immunoreactive to anti-synapsin and where the dendrites of Kenyon cells form synapses with axon terminals of projection neurons (Fig 4G–4I). The vertical lobe is innervated by TH-positive fine fibers, mainly the distal to medial portion (Fig 4D). Immunoreactive fibers appear to run perpendicularly to the Kenyon cell axons (Fig 4D). The medial lobe also receives TH-positive fibers (Fig 4M), but immunonegative area as in dopamine immunolabeling is not visible. TH-immunoreactive signals are more prominent near the tip (arrows in Fig 4M). In the inferior medial protocerebrum anterior to the medial lobe, the DIP1 cell cluster is located (triangles in Fig 4M).

### Neurobiotin injection into a distal region of the vertical lobe

To confirm that the dopamine/TH-positive DCa1 neurons (Figs 3J and 4J) project fibers to the vertical lobe, we injected a neurotracer Neurobiotin into a distal region of the vertical lobe. Neurobiotin injection into the tip of the vertical lobe (see asterisk in Fig 5A for injection site) revealed the following four groups of neurons innervating the vertical lobe: 1) neurons with cell bodies lateral to the vertical lobe (arrowheads in Fig 5A); 2) neurons with cell bodies ventral to the lateral calyx (arrows in Fig 5B); 3) a neuron with its cell body ventral to the medial calyx (triangle in Fig 5B); and 4) Kenyon cells (small arrowheads in Fig 5B). The second group corresponds exactly to the DCa1 neurons according to their cell body locations and axon projections, supporting the interpretation that the dopaminergic DCa1 neurons innervate the distal region of the vertical lobe. Neither DCa2 nor DSP1 neurons were filled by Neurobiotin injection.

### Gross anatomy of the central complex and associated neuropils

**Fig 6:**
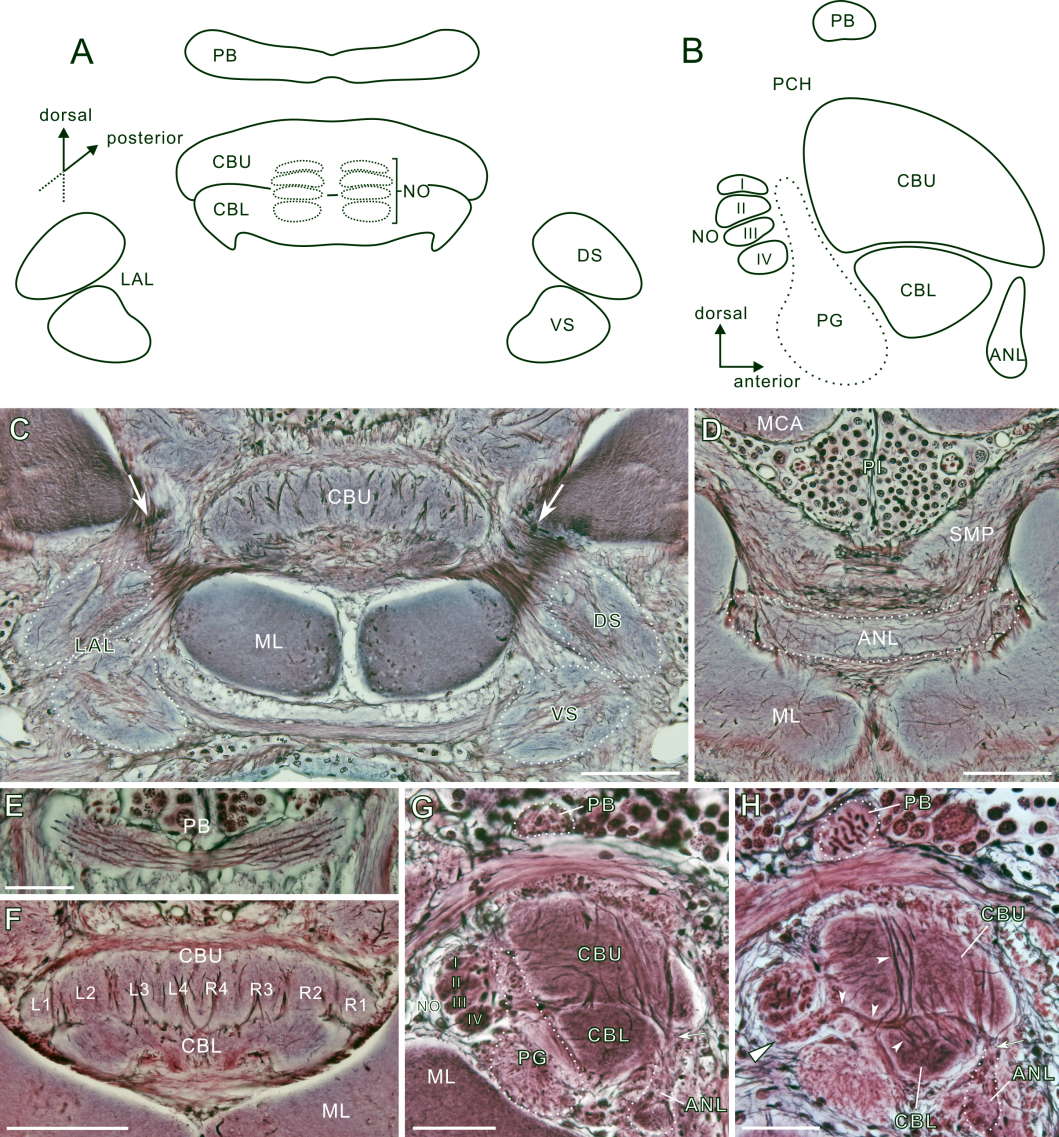
Structure of the central complex and associated neuropils. **A**: Diagram of a frontal section of the central complex and lateral accessory lobe (LAL), the latter further subdivided into the dorsal shell (DS) and ventral shell (VS). Dotted lines indicate a pair of globular noduli (NO) posterior to the central body (CB). The central body comprises the upper division (CBU) and the lower division (CBL). Each nodulus consists of four subunits (I-IV, see panel B). **B**: Diagram of a sagittal section of the central complex. The protocerebral bridge (PB) is situated above the central body. The anterior lip (ANL) is located ventro-anteriorly to the lower division of the central body. C-G: Bodian-stained brain sections. **C**: Frontal section through the upper division of the central body and the lateral accessory lobes. Arrows indicate fibers connecting the central body and the lateral accessory lobe. **D**: Frontal section through the anterior lip and the medial lobe (ML). **E**: Frontal sections through the protocerebral bridge. Longitudinal fibers span the midline. **F**: Frontal section through the central body. The upper division is divided into eight subunits called slices, four per hemisphere. **G, H**: Sagittal sections through the central body. A bundle of fibers connects a subunit IV of the nodulus (triangle) to lower division of the central body (arrowheads in H). PCH, posterior chiasma; PG, posterior groove; SMP, superior medial protocerebrum. Scale bars = 100 μm in C, D, F; 50 μm in E, G, H.

Fig 6 shows the anatomical organization of the central complex of *P*. *americana*. The terminology is based on that for the locust, *Schistocerca gregaria* [57,58], and also on the recently updated nomenclature for insect brains [59]. The central complex is a modular midline neuropil in the protocerebrum, situated between the paired pedunculi and dorsal to the medial lobes of the mushroom body. The central complex consists of three prominent structures, the protocerebral bridge (PB), the central body (CB) and the paired noduli (NO) (Fig 6A and 6B). The central body comprises the upper and lower divisions (CBU and CBL, respectively) (Fig 6A and 6B). The protocerebral bridge is a curvilinear rod-like midline-spanning neuropil in the superior protocerebrum beneath the pars intercerebralis (Fig 6A and 6E). Two hemispheres of the bridge are connected by commissural fibers and appear to be continuous across the midline (Fig 6E). The central body lies below the protocerebral bridge. The central body is a lip-shaped structure in *P*. *americana*, the upper division covering the smaller lower division (Fig 6F). The upper division of the central body is arranged in repetitive structural subunits termed slices [59], and at least four slices are distinguished in each hemisphere (L1-4 or R1-4) while such columnar organization is not visible in the lower division (Fig 6F). A small neuropil, termed the anterior lip (ANL), is situated in front of the lower division of the central body (Fig 6B, 6G and 6H). The paired noduli are located posterior to the central body, each consisting of four subunits (I-IV) (Fig 6B and 6G). A bundle of fibers (arrowheads in Fig 6H), which bypasses the upper division of the central body, connects the lower division of the central body and subunit IV of the nodulus (triangle in Fig 6H). Ventro-laterally, the central body is connected to the lateral accessory lobe (LAL) (arrows in Fig 6C). The lateral accessory lobe is composed of two oval structures, the dorsal shell (DS) and ventral shell (VS) (Fig 6A and 6C).

### Dopamine immunoreactivity in the central complex and the lateral accessory lobe

**Fig 7:**
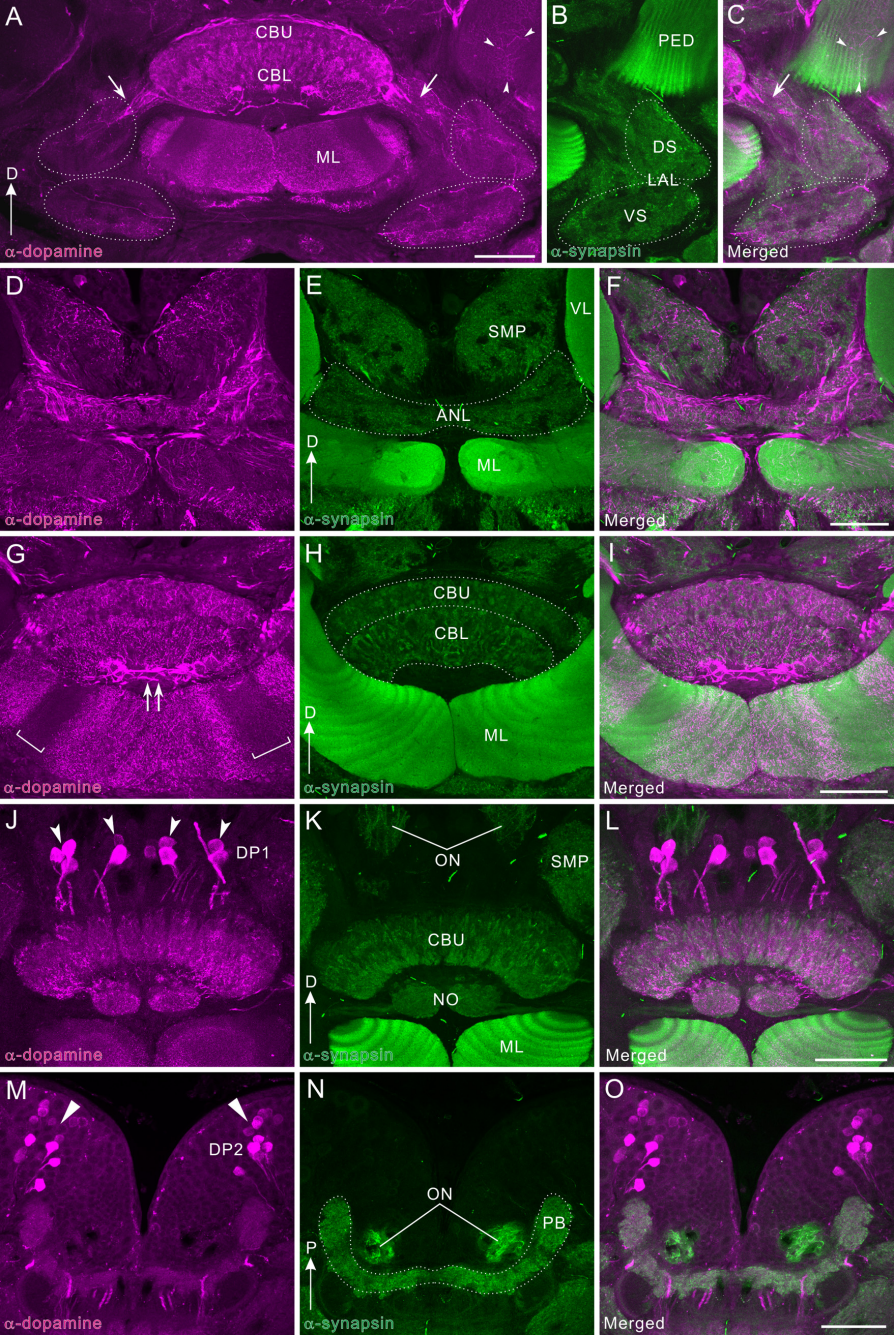
Dopamine immunoreactivity in the central complex and lateral accessory lobe. A-L: Frontal sections. M-O: Horizontal sections. Dopamine immunoreactivity shown in magenta (A, D, G, J, M) and synapsin immunoreactivity in green (B, E, H, K, N). Corresponding merged images in C, F, I, L, O. **A-C**: Frontal sections through the central body (CB) and lateral accessory lobe (LAL). The upper and lower divisions of the central body (CBU and CBL, respectively) display intense dopamine immunoreactivity. Immunoreactive fibers innervate the dorsal and ventral shell of the lateral accessory lobe (DS and VS, respectively) and parts of the pedunculus (PED; arrowheads in A,C). A bundle of fibers connecting the central body to the lateral accessory lobe also exhibits dopamine immunoreactivity (arrows in A,C). **D-F**: Frontal sections through the anterior lip (ANL) innervated by dense dopamine-immunoreactive fibers. Labeled fibers are also seen in the superior medial protocerebral neuropil (SMP). **G-I**: Frontal sections through the central body and medial lobe (ML). Immunoreactive fibers are widely distributed in both structures, except for part of the medial lobe indicated by brackets in G. The immuno-negative region is flanked by a dense meshwork of dopamine-immunoreactive fibers. Double arrow in G indicates a plexus of immunoreactive processes in the posterior groove, originating from DP2 neurons. **J-L**: Frontal sections through the posterior portion of the central body. DP1 cell bodies (arrowheads) extend their axons ventrally, and a part of the noduli (NO) display dopamine immunoreactivity. **M-O**: Horizontal sections through the protocerebral bridge (PB). DP2 clusters (indicated by triangles) extend their axons anteriorly. Immunoreactive fibers bypass the protocerebral bridge, which lacks dopamine immunolabeling. D, dorsal; ON, ocelluar nerve; P, posterior. Scale bars = 100 μm.

**Fig 8:**
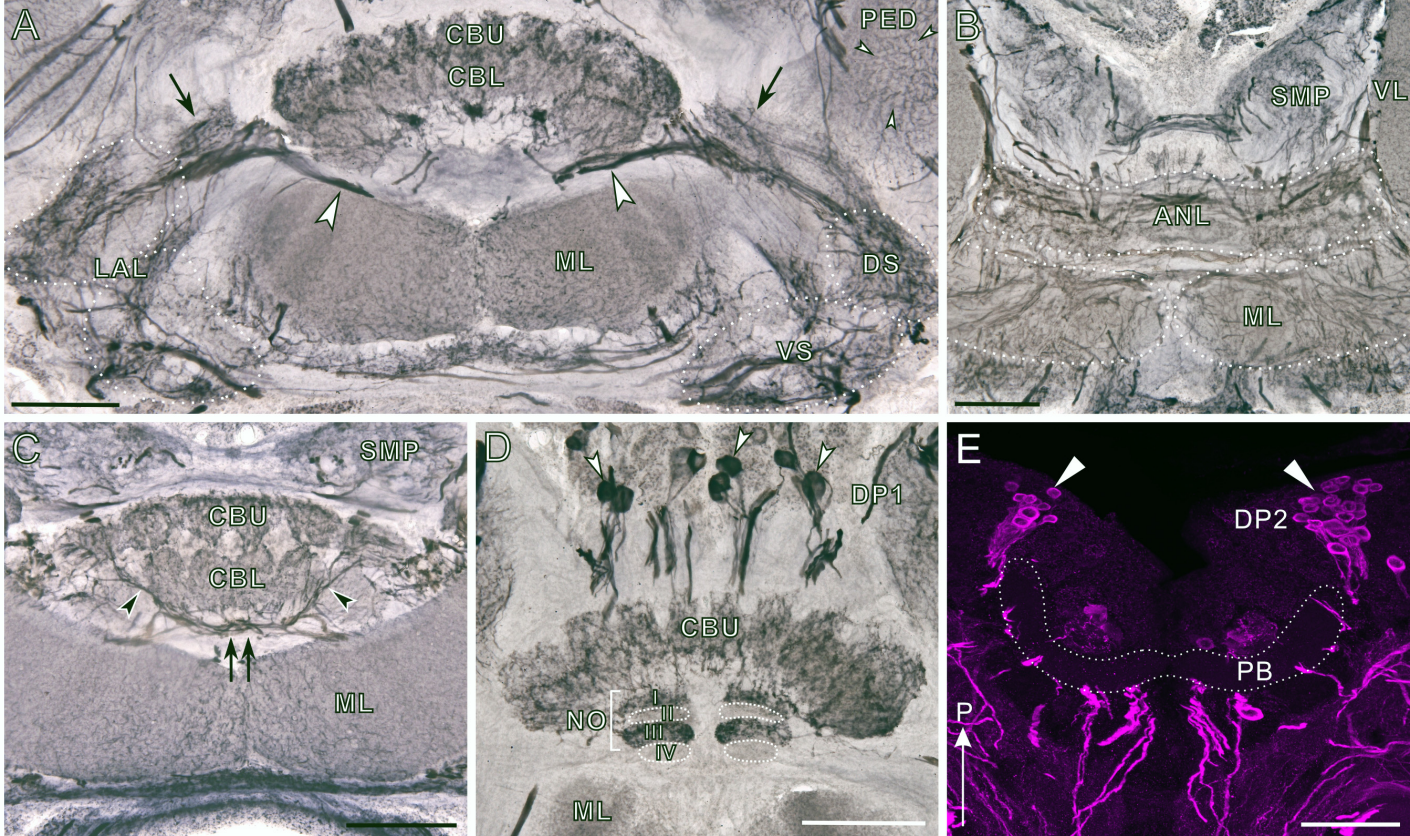
Tyrosine hydroxylase immunoreactivity in the central complex and the lateral accessory lobe. A-D: Frontal sections (immunoperoxidase preparations, dorsal to the top). E: Horizontal section (immunofluorescent preparation, posterior to the top). **A**: Frontal section through the central body (CB) and the lateral accessory lobe (LAL). Tyrosine hydroxylase (TH)-positive fibers innervate the upper and lower divisions of the central body (CBU and CBL, respectively). A bundle of immunoreactive fibers (arrows) connects the central body to the lateral accessory lobes comprising dorsal and ventral shells (DS and VS, respectively). The dorsal and ventral shells are also innervated by TH-immunoreactive fibers. Moderately immunolabeled fibers are visible in the pedunculus (PED, small white arrowheads). Large arrowheads indicate immunoreactive axons originating from DP2 neurons (see Fig 11). **B**: Frontal section through the anterior lip (ANL), innervated by a plexus of TH-positive fibers. Immunoreactive fibers are also seen in the superior medial protocerebral neuropil (SMP) and the medial lobe (ML). **C**: Frontal section through the central body, posterior to the section in panel A. Immunoreactive fibers in the posterior groove (double arrow) that originate in DP2 neurons invade the central body to innervate this neuropil (arrowheads). The medial lobe (ML), especially the distal portion, receives numerous fine immunoreactive fibers. **D**: Frontal section through a posterior portion of the central body. DP1 cell bodies (arrowheads) extend their axons ventrally. Subunits I and III of the noduli (NO) exhibit TH immunoreactivity whereas II and IV lack immunolabeling except for faint innervation in lateral part of the subunit II. **E**: Horizontal section through the protocerebral bridge (PB). DP2 cell clusters are located posterior to the protocerebral bridge, which is free of immunoreactivity. P, posterior. Scale bars = 100 μm.

All subdivisions of the central complex excluding a part of the noduli and the protocerebral bridge exhibit moderate to strong dopamine immunoreactivity (Fig 7). The dorsal and ventral shell of the lateral accessory lobes are innervated by a sparse meshwork of dopamine-immunoreactive fibers, which are connected to the central body (arrows in Fig 7A). The anterior lip is densely innervated by a plexus of thick dopamine-immunoreactive fibers as well as beaded fine processes (Fig 7D). The central body exhibits intense dopamine immunoreactivity (Fig 7G). The upper division is innervated by fine immunoreactive fibers while the lower division exhibits a granular pattern of labeling (Fig 7G). A plexus of fibers in the posterior groove exhibits strong dopamine immunoreactivity (double arrow in Fig 7G). A part of the noduli exhibit granular immunoreactivity (Fig 7J). Above the central body, a group of cell bodies (DP1) are immunolabeled (arrowheads in Fig 7J). The DP1 neurons do not invade the central body, and instead the axons run in a region posterior to the central body. Posterior to the dopamine-negative protocerebral bridge, a cluster of immunopositive cell bodies (DP2) are located (triangles in Fig 7M). DP2 neurons project their axons ventrally, which bifurcate on the way to innervate the central body and the lateral accessory lobe (see Fig 8A, 8C and 8E).

### TH immunoreactivity in the central complex and the lateral accessory lobe

Corresponding to the dopamine immunolabeling, TH-immunoreactive fibers innervate all subdivisions of the central complex excluding the protocerebral bridge and part of the noduli. In the lateral accessory lobe, the constituent dorsal and ventral shell are sparsely innervated by strongly labeled TH-positive fibers (Fig 8A). In the anterior lip, numerous fibers exhibit moderate TH immunoreactivities (Fig 8B). The central body is densely innervated by TH-immunoreactive processes (Fig 8A and 8C). TH-immunoreactive processes in the posterior groove (double arrow in Fig 8C) invade the lower and upper division of the central body to innervate these neuropils (arrowheads in Fig 8C). These immunoreactive processes are attributed to DP2 cell bodies (Fig 8E). In the noduli, subunits I and III exhibit a granular pattern of TH immunolabeling while subunits II and IV lack immunoreactivity except for faint innervation in lateral part of the subunit II (Fig 8D). Also as in dopamine immunolabeling, DP1 cell bodies were labeled by the anti-TH dorsal to the central body (arrowheads in Fig 8D). Likewise, DP2 neurons (triangles in Fig 8E) are immunolabeled in a region postero-laterally from the TH-negative protocerebral bridge.

### Dopamine immunoreactivity in the deutocerebrum

**Fig 9:**
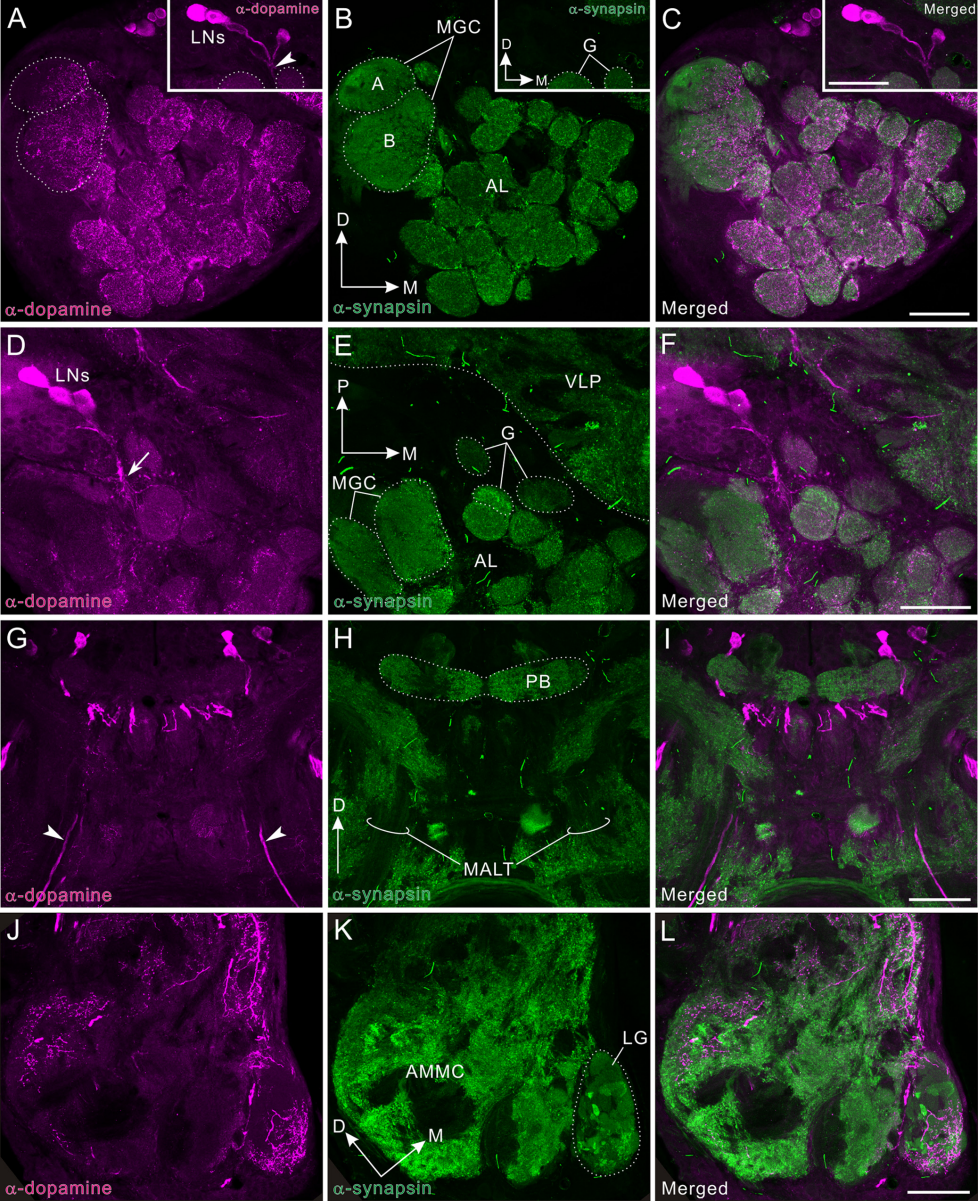
Dopamine immunoreactivity in the deutocerebrum and medial antennal lobe tract. Dopamine immunoreactivity is shown in magenta (A, D, G, J) and synapsin immunoreactivity in green (B, E, H, K). Corresponding merged images (C, F, I, L). **A-C**: Frontal sections though the antennal lobe (AL). Antennal lobe glomeruli (G in inset of B) are innervated by moderately immunolabeled fine fibers, which arise from local interneurons (LNs in inset of A) of the DAL cell cluster. **D-F**: Horizontal sections through the antennal lobe. Dopamine-immunoreactive local interneurons arborize to bear fine fibers (arrow). **G-I**: Frontal sections through the medial antennal lobe tract (MALT) containing a pair of dopamine-positive fibers (arrowheads). **J-L**: Frontal sections though the lobus glomerulatus (LG) and antennal mechanosensory and motor center (AMMC). A dorso-lateral part of the AMMC, and ventral and dorso-medial region of the lobus glomerulatus receive dopamine-positive fibers. D, dorsal; M, medial; P, posterior. Scale bars = 100 μm.

The antennal lobe exhibits moderate dopamine immunoreactivity, which is attributed to the antennal lobe local interneurons (Fig 9A and 9D). In the antennal lobe, all ordinary glomeruli involved in processing general odors exhibit a uniform finely granular immunolabeling pattern (Fig 9A). The macroglomerular complex (MGC in Fig 9B), which consists of two enlarged glomeruli (glomerulus A and B) and is specialized for sex pheromone processing, also displays granular immunolabeling (Fig 9A). In these glomeruli, signals are not uniform, but instead are more concentrated at the medial side (Fig 9A). A bilateral pair of fibers exhibits dopamine immunoreactivity in the medial antennal lobe tract (arrowheads in Fig 9G), but their origin has not been confirmed. A connection via the medial antennal lobe tract to the calyces and the lateral horn was not confirmed. Dopamine-immunoreactive fibers also innervate the lobus glomerulatus (LG) and the antennal mechanosensory and motor center (AMMC). In the lobus glomerulatus, blebbed dopamine-immunoreactive fibers innervate the ventral and dorso-medial region (Fig 9J). In the AMMC, beaded immunoreactive fibers exclusively innervate the dorso-lateral regions (Fig 9J).

### TH immunoreactivity in the deutocerebrum

**Fig 10:**
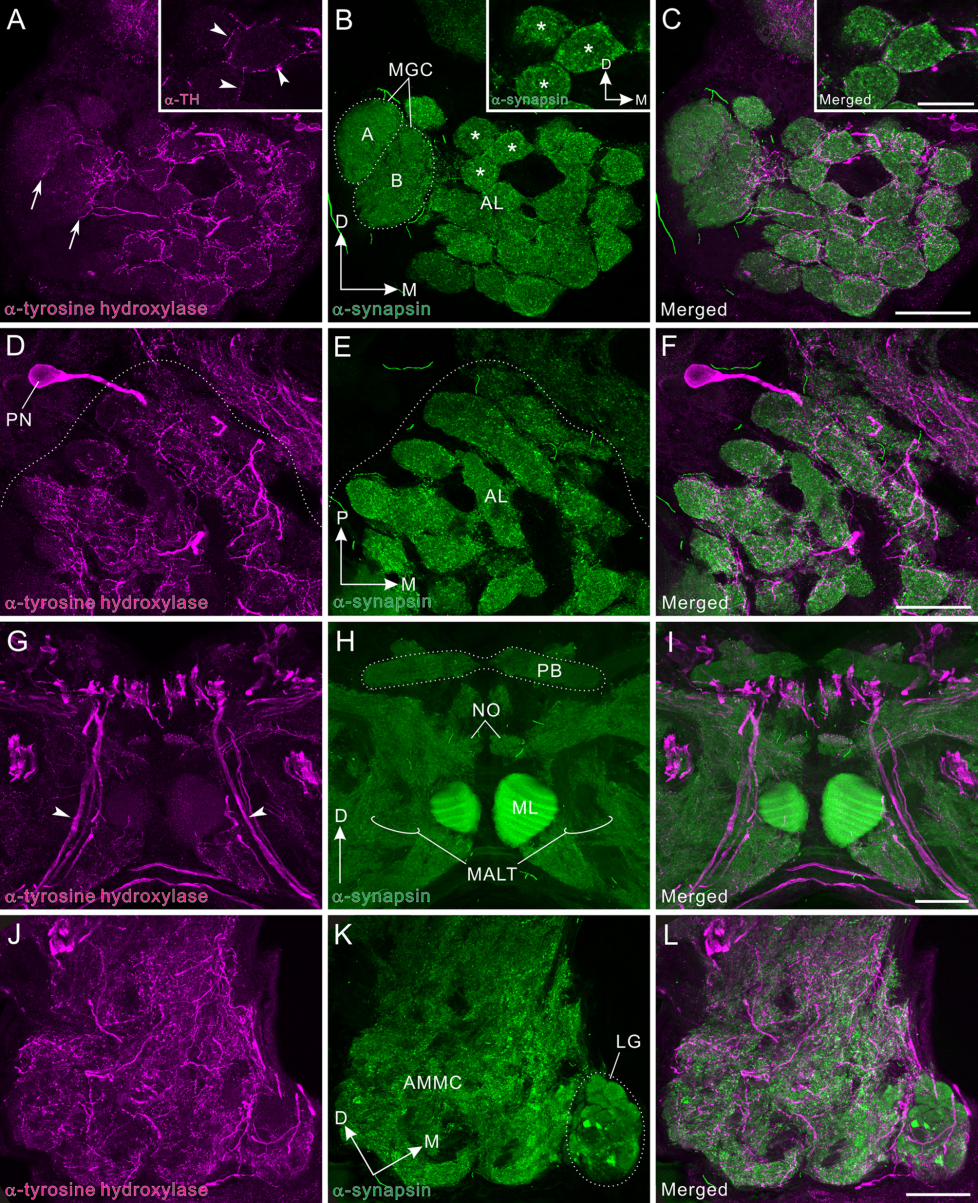
Tyrosine hydroxylase immunoreactivity in the deutocerebrum and medial antennal lobe tract. Tyrosine hydroxylase (TH) immunoreactivity is shown in magenta (A, D, G, J) and synapsin immunoreactivity in green (B, E, H, K). Corresponding merged images (C, F, I, L). **A-C**: Frontal sections through the antennal lobe (AL). Insets indicate single optical slice (< 2 μm thickness) of the three glomeruli, which are marked by asterisks in main panel B. TH-positive fibers innervate antennal lobe glomeruli, predominantly the peripheral areas (see arrowheads in inset of A). In the macroglomerular complex (MGC), TH-positive fibers innervate exclusively the medial side (arrows in A). **D-F**: Horizontal sections through the antennal lobe. A single TH-positive multiglomerular projection neuron (PN) is labeled, which projects its axon toward the medial antennal lobe tract (MALT, see also panel G). **G-I**: Frontal sections through the medial antennal lobe tract containing several TH-positive axons (arrowheads). **J-L**: Frontal sections through the lobus glomerulatus (LG) and the antennal mechanosensory and motor center (AMMC). TH-immunoreactive fibers are widely distributed in the AMMC. In the lobus glomerulatus, immunoreactive fibers predominantly innervate the ventral portion. D, dorsal; M, medial; ML, medial lobe; NO, noduli; P, posterior; PB, protocerebral bridge. Scale bars = 50 μm in inset of C; 100 μm in C, F, I, L.

**Fig 11:**
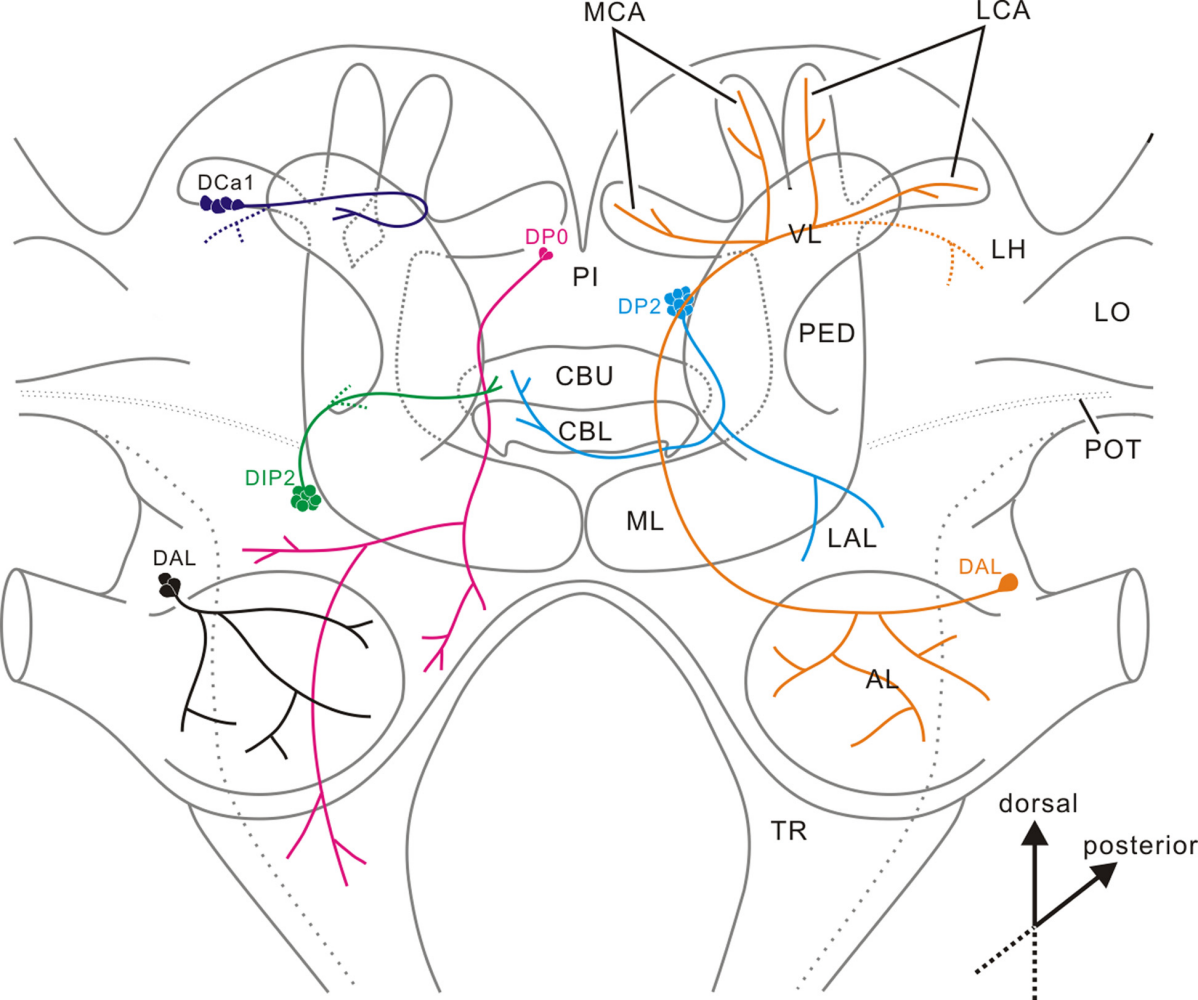
Diagram depicting cell body locations and the principal fiber trajectory of dopaminergic neurons in the American cockroach. Solid lines indicate confirmed pathways, and dotted lines in color are putative pathways. All abbreviations for neuropil structures are as in Fig 2.

TH-immunoreactive fibrous processes widely innervate the antennal lobe glomeruli (Fig 10A). However, the distribution pattern and origin of these neurites differs totally from those of dopamine-immunoreactive fibers (Fig 10A–10F). The anti-TH antiserum immunolabeled a bilateral pair of multiglomerular projection neurons, but did not any antennal lobe local interneurons (Figs 10D and 11). The multiglomerular TH-immunoreactive fibers in the ordinary glomeruli innervate predominantly the periphery (Fig 10A–10C), which is more obvious in single optical slices (insets in Fig 10A–10C). In the macroglomeruli (both glomerulus A and B), TH-immunoreactive fibers innervate the very medial margins (arrows in Fig 10A). In the medial antennal lobe tract, in which axons of projection neurons run, a few axons exhibit TH immunoreactivity (arrowheads in Fig 10G). Among these, a pair of axons belongs to TH-immunoreactive multiglomerular projection neurons while others may be descending fibers. In the AMMC, TH-immunoreactive processes are widely distributed (Fig 10J). In the lobus glomerulatus, TH-immunoreactive processes mainly innervate the ventral half (Fig 10J–10L) as seen in dopamine immunolabeling (Fig 10J–10L).

## Discussion

In the supraesophageal ganglia without the optic lobes (called the cerebrum [59]) of the cockroach, *Periplaneta americana*, we identified ~ 250 dopamine-immunoreactive cell bodies per hemisphere, which constitute at least 15 classes of neurons (Fig 2 and Table 2). The number of dopamine-immunoreactive neurons is greater than that in the desert locust (ca. 100) and blowfly (ca. 150), and in contrast slightly lower than that in the honeybee (ca. 310) [9–11]. Even though the number of dopaminergic neurons is rather small compared with the total number of cells in the brain, their processes are widely distributed throughout the brain. The wide distribution of neuronal processes from dopaminergic neurons is similar to other neurons releasing different amines, e.g. octopamine [60,61] and serotonin [62,63] in insects. Such anatomical features are well suited to affect the activity of major brain regions.

In addition to the anti-dopamine antiserum, we also employed an independent marker for dopaminergic neurons, a tyrosine hydroxylase (TH) antiserum. These two antisera immunolabeled almost the same groups of neurons with a notable discrepancy in the antennal lobe: local interneurons were immunolabeled by the dopamine antiserum (Fig 9A and 9D) as reported by Distler (1990), while a bilateral pair of projection neurons was also labeled with anti-TH (Fig 10A and 10D). It is not likely that the anti-TH non-specifically binds a protein other than TH because 1) the antiserum recognized only a single band of protein in a Western blot (Fig 1), and moreover 2) the molecular size of this band is close to that of *D*. *melanogaster* TH [49]. The concentration of TH in the dopamine-positive local interneurons can be very low. Dopamine in TH-positive projection neurons might be concentrated in axons (Fig 9G) and release sites (calyces; Fig 3G and 3G’), or alternatively rapidly metabolized to other molecules. Another possibility is that dopamine biosynthesis in the projection neurons is context-dependent as reported in octopamine synthesis in locust [61,64]. They might actively produce dopamine only in certain occasion, and otherwise they do not. For some groups of dopaminergic neurons, we could follow their axon trajectories in serial sections. Fig 11 summarizes the projection patterns of six classes of dopaminergic cell groups. For this purpose, we relied on TH-immunolabeling because the anti-TH antiserum provided more intense signals especially in axons and fibers compared with the anti-dopamine antiserum.

### The mushroom body

The mushroom body of the cockroach is substantially supplied with the dopamine-immunoreactive fibers, suggesting that dopamine engages in fundamental tasks of this neuropil area. The mushroom body of *P*. *americana* contains ca. 200,000 intrinsic Kenyon cells per hemisphere [65]. This is the largest number ever reported in an insect. For comparison, that of honey bee comprises 170,000 Kenyon cells [66]. The Kenyon cells of *P*. *americana* largely fall into three types (class I—III) [53,67]. Class I can be further subdivided into three morphologically different subtypes (K1-3) [54]. The Kenyon cells not only receive predominantly olfactory inputs via the projection neurons, that are postsynaptic in turn to olfactory receptors in the antennal lobe glomeruli, but also visual inputs [68,69]. Several lines of evidence suggest that the mushroom body plays important roles in odor information processing [32,33], and in the formation of olfactory and visual place memory [34,36]. Dendrites of the Kenyon cells ramify in the paired calyx. The calyx receives a fibrous meshwork of dopaminergic processes (Figs 3G’ and 4G). These can be attributed to a bilateral pair of projection neurons revealed by TH immunolabeling (Figs 10D and 11). This is the first anatomical evidence suggesting that an antennal-lobe projection neuron produces dopamine. The Kenyon cell axons pass through the pedunculus to bifurcate at the base; one projecting into the vertical lobe and the other into the medial lobe [48]. The Kenyon cells form neural connections with extrinsic (output) neurons in the pedunculus and lobes. The pedunculus and lobes consist of clearly repetitive longitudinal modular subunits called either slabs or laminae, about 15 dark and 15 pale slabs, which refer to their appearance in silver-stained preparations, being alternatively stacked [48,53]. Dopaminergic fibers invade the medial and vertical lobes, predominantly the γ layers, from the anterior face (Figs 3J, 3M, 4J and 4M). Such a pattern of innervation occurs predominately in distal portions of the lobes. These immunoreactive fibers might modulate neural connections between Kenyon cell axons and the dendrites of output neurons innervating the distal lobes. In the cricket, dopaminergic neurons are critical neural substrates for the formation and retrieval of aversive memory, but not for those of appetitive memory, in both olfactory and visual learning [7,70–73]. Likewise, dopaminergic systems also mediate aversive olfactory learning in the honeybee [74], although their participation in retrieval has yet to be demonstrated. In the honeybee, a group of dopamine-immunoreactive neurons with cell bodies beneath the lateral calyx (a subset of C_3_ neurons) project their fibers to the vertical lobe [9]. Also in the fruit fly, a group of dopaminergic neurons with cell bodies lateral to the calyx innervate the vertical lobe and the spur of the mushroom body (PPL1) [75]. The PPL1 neurons are anatomically very similar to the cockroach DCa1 neurons innervating the vertical lobe (Figs 3J, 4J and 11). Because the PPL1 neurons in the fruit fly mediate aversive reinforcement in both olfactory and visual learning [76,77], cockroach DCa1 neurons might also be engaged in aversive memory formation. In addition, in the fruit fly a subset of another group of dopaminergic (PAM) neurons with cell bodies in an anterior medial region of the protocerebrum and axons projecting into the medial lobes, conveys reward signals in olfactory and visual learning [76,78]. In the corresponding region of the cockroach brain, exists a set of dopaminergic cell bodies (DIP1) (Figs 2, 3M and 4M). The DIP1 may be homologous to the PAM neurons according to the location of their cell bodies and their innervation of the medial lobe. It is an intriguing question whether or not DIP1 neurons are involved in reward learning.

### The central complex and associated neuropils

The central complex of the cockroach is innervated by numerous dopaminergic fibers, suggesting that dopamine plays important roles in this neuropil. The central complex is a conspicuous midline-spanning neuropil with higher-order visual inputs [79,80]. This brain structure is involved in several functions, such as motor control, spatial orientation, visual spatial memory, and also various forms of arousal [79]. In the cockroach, its roles in locomotor control have been characterized electrophysiologically [38,39]. Despite such vital roles, the detailed architecture of the central complex and its associated neuropils have not been clearly described in the cockroach. Reduced silver impregnation reveals that the structural organization in the cockroach (Fig 6A and 6B) is similar to that in the desert locust [57,58,81]. The central complex of *P*. *americana* consists of the rod-like protocerebral bridge and a lip-like central body; its upper division covers the lower division, and both are connected to the ventro-laterally located lateral accessory lobe comprising dorsal and ventral shells (Fig 6C). The upper division of the central body is internally organized into 8 subunits (called slices [59]), 4 in each hemisphere (Fig 6F), as implied in proctolin immunolabeling [82]. An internal layered organization as in the desert locust [57], monarch butterfly [83], and fruit fly [59] was recognized neither in the upper division nor in the lower division of the central body (Fig 6). To visualize them, specific antibody markers would be needed. Posterior to the central body, a pair of globular noduli is found, which are composed of four subunits as in other insects [59,81,84]. Subunit IV is connected to the lower division of the central body (Fig 6H), probably corresponding to the lower unit of the nodulus in the desert locust.

In the fruit fly, a series of studies using mutants with structural defects in different compartments of the central complex and gene expression targeted by the Gal4/UAS system have revealed roles in visual spatial memory, and different areas of the central body are involved in different learning paradigms [79]. So far, however, the involvement of dopaminergic neurons has not been reported in visual spatial memory. Another important function of the central complex is that as a motor control center for walking, flight, acoustic communication, and courtship. In the cockroach [38,39] and fly [82,85], the central complex participates in the control of walking activity as well as in fine tuning motor patterns. Accordingly, in the fruit fly various forms of arousal and activity states are mediated by the central complex, mostly via dopaminergic systems [79]. One of the dopaminergic neurons, the function and arborization pattern of which is well-characterized, is a fan-shaped body tangential neuron PPM3 in the fruit fly, and these neurons modulate aggressive behavior [86]. DP2 neurons (Figs 7M, 8E and 11) in the cockroach may be homologous to PPM3 according to their cell body locations and projection patterns. Whether and, if so, how DP2 neurons are involved in mediating aggressive behavior of the cockroach will need to be addressed in the future.

### Comparative aspects of dopaminergic neurons

Comprehensive characterization of putatively dopaminergic neurons has been achieved in the honeybee [9], locust [11], blowfly [10], fruit fly [12], and now the cockroach (present study). Although there are minor differences, the dopaminergic neurons in the brain of these insects appear to be conserved, especially in neurons innervating the mushroom body and the central complex. For instance, the DCa1 neurons of the cockroach show a close resemblance to a subset of C_3_ neurons in the honeybee [9] and the PPL1 neurons in flies [10,75]. Their cell bodies are located in the cell body rind ventro-lateral to the calyx and extend their axons into the vertical lobes. However, corresponding neurons have so far not been reported in the locust. In addition, the small cell bodies in the inferior medial protocerebrum also show close resemblance among insects. They innervate the mushroom body lobes and/or the surrounding protocerebral neuropils: C_1_ neurons in the honeybee [9], DIP1 neurons in the locust [11], PAM neurons in flies [10,12], and DIP1 neurons in the cockroach (present study). In addition, DIP2 neurons in the cockroach, C_2_ neurons in the honeybee [9], and DIP2 neurons in the locust [11] may also be homologous. In terms of neurons innervating the central body, the DP2 neurons of the cockroach are rather similar to a subset of Sp neurons in the honeybee [9], DP2 neurons in the locust [11], and PPM3 neurons in flies [10,12].

On the other hand, there are considerable species-specific differences in the antennal lobe. In the cockroach and honeybee, either local interneurons or deutocerebral neurons [9,24] innervate the antennal lobe glomeruli, while the antennal lobe of both the locust and flies completely lacks dopaminergic fibers [10–12]. In the cockroach, not only local interneurons but also a bilateral pair of multiglomerular projection neurons, the latter being revealed by TH immunolabeling, seem to produce dopamine. So far, no equivalent for this type of neuron has been reported in other insects.

### Functional roles of dopamine neurons

Dopamine’s actions in the cockroach nervous system are probably diverse, ranging from a role as a neurotransmitter and as a neuromodulator, as implied in this account as well as the previous ones [10,11,87]. In the peripheral systems of the cockroach, dopamine functions as a transmitter for a salivary neuron (SN1), which induces the secretion of protein-free saliva [16]. In the brain, dopamine appears to act from various types of interneuron either with small- or wide field arborizations.

In many insects so far studied, dopamine provides an aversive value to sensory stimuli such as odor, color and visual patterns that are neutral before they are associated with aversion [5,6,74,76]. This may also be the case in the cockroach, but behavioral and physiological experiments combined with pharmacology are definitely needed to demonstrate this possibility. Several lines of evidence suggest that aversive reinforcement, especially in olfactory memory, is based on increased efficiency of synaptic transmission between Kenyon cells that represent a particular conditioned stimulus and the corresponding mushroom body output neurons, and that this is mediated by dopamine exocytosis [88,89]. In the fruit fly, the presentation of a conditioned odor, followed by a transient activation of a subset of dopaminergic PPL1 neurons innervating the vertical lobe, induces aversive olfactory memory, so that the aversive stimulus can be substituted by activation of PPL1 neurons [76]. As just described, through the last decade, important neurons engaged in learning and memory have been characterized in *D*. *melanogaster*. However, the dynamics of reinforcement is still poorly understood at the single cell level. The cockroach may be an ideal species to monitor the dynamic range of plasticity of single neurons because unlike *D*. *melanogaster* they are quite amenable to intracellular electrophysiological recording methods. The DCa1 neurons that are anatomically equivalent to the fruit fly’s PPL1 neurons are probably the most promising candidate to address physiologically the role of dopaminergic systems in the brain, in particular, the neural mechanism underlying aversive reinforcement.
